# Potential value of T1 mapping in cardiac MR assessment of hypertrophic cardiomyopathy and dilated cardiomyopathy patients: preliminary results

**DOI:** 10.1186/1532-429X-14-S1-P145

**Published:** 2012-02-01

**Authors:** Alexis Jacquier, Alexandros Kallifatidis, Franck Thuny, Jean Michel Bartoli, Boris Maurel, Guy Moulin

**Affiliations:** 1 CEMEREM, UMR 6612 CNRS, Marseille, France; 2 Radiology, CHU Timone, Marseille, France; 3 Cardiology, CHU Timone, Marseille, France

## Summary

The purpose of this study is to assess the potential value of T1 mapping in assessing fibrosis in hypertrophic cardiomyopathy (HCM) and dilated cardiomyopathy (DCM) in comparison to late contrast enhanced imaging using cardiac MR.

## Background

Myocardial fibrosis causes 1) increased stiffness and induces pathological signaling in cardiomyocytes resulting in progressive cardiac failure, 2)impairs mechano-electric coupling of cardiomyocytes and increases the risk of arrhythmias. T1 mapping allow to quantify the total amount of myocardial extracellular volume and to subsequently define the amount of myocardial fibrosis. Quantification of fibrosis in dilated and hypertrophic cardiomyopathies and it's link to delayed myocardial enhancement is unclear.

## Methods

Twelve patients with HCM, 10 with DCM, and 5 controls subjects were prospectively included. All patients underwent cardiac MR with the following sequences (parameters set according to recommendation of the SCMR): 1) cine sequence, 2) Molli sequence (TR/TE=3.0ms/1.5ms; matrix 144x150, thickness:7 mm) acquired before and 5, 7, 9 min after gadolinium injection (DOTAREM, 0,2mmol/kg). 3) Late gadolinium enhanced sequence 10min after injection. T1 values were assessed in blood and in the 6 segments of the mid LV slice. Patient with (+) and without (-) enhancement on LGE were analysed separately. Were calculated: R1, ΔR1, ΔR1 ratio (partition coefficient of Gd, λ).

## Results

**Figure 1:**
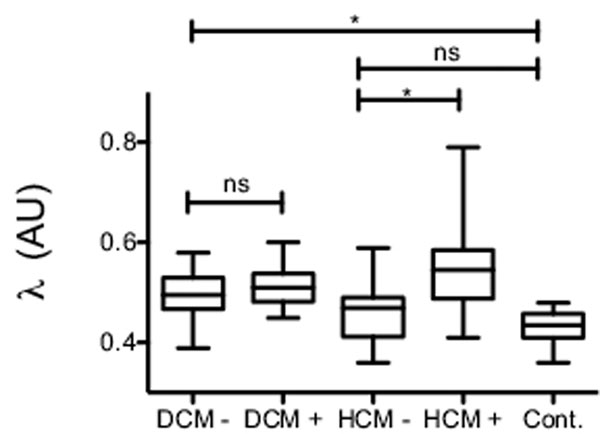
Graph showing the λ value from different group.

in controls before injection T1 value was 0,98s ±0,05 s in myocardium, and 1,63 s ±0,08 in blood. λ was measured at 0.43±0,03 in normal. In DCM, λ was not significantly different between patient with (0.51±0,04) and without (0.50±0,05) enhancement on LGE (p=0.2). In HCM, λ was significantly different between patient with (0.54±0,08) and without (0.45±0,05) enhancement on LGE (p<0.0001)(figure 1). No significant difference was measured in λ value between control and HCM - (p=0.1).

## Conclusions

T1 mapping allow for quantification of the total amount of fibrosis in a myocardium. In DCM the distribution of fibrosis might be more diffused compared with HCM. These results have to be confirmed by larger study.

## Funding

PHRC national 2011, AORC excelence 2011.

